# No difference in knee muscle activation and kinematics during treadmill walking between adolescent girls with and without asymptomatic Generalised Joint Hypermobility

**DOI:** 10.1186/s12891-021-04018-w

**Published:** 2021-02-11

**Authors:** Helene Nikolajsen, Birgit Juul-Kristensen, Peter Fjeldstad Hendriksen, Bente Rona Jensen

**Affiliations:** 1 Research Unit of Applied Health Science, University College South Denmark, Lembckesvej 7, DK-6100 Haderslev, Denmark; 2 Research Unit of Musculoskeletal Function and Physiotherapy, Department of Sports Science and Clinical Biomechanics, University of Southern Denmark, Campusvej 55, DK-5230 Odense M, Denmark; 3 National Research Centre for the Working Environment, Lersø Parkallé 105, DK-2100 Copenhagen, Denmark; 4 Department of Neurology, Odense University Hospital, University of Southern Denmark, Sdr. Boulevard 29, 5000 Odense, Denmark; 5 Department of Clinical Research, University of Southern Denmark, Winsløwparken 19, 5000 Odense, Denmark

**Keywords:** Gait, Treadmill walking, Generalized Joint Hypermobility, Kinematics, Kinetics, Electromyography (EMG)

## Abstract

**Background:**

Altered knee muscle activity in children with asymptomatic Generalized Joint Hypermobility (GJH) is reported during isometric contraction, static and dynamic balance tasks and jumping, but has not been studied during gait. Therefore, the aim was to investigate group differences in knee muscle activity simultaneously with knee joint kinematics during treadmill walking between children with and without GJH.

**Methods:**

Girls 14–15 years of age with GJH (inclusion criteria: Beighton score ≥6 of 9 and positive hyperextension ≥10° (one/both knees)) and a matched control group without GJH (inclusion criteria: Beighton score ≤5 and no knee hyperextension ≥10° ) were recruited. In total 16 participants with GJH and 10 non-GJH participants were included in the study.

Surface electromyography (sEMG) was measured from the quadriceps, hamstrings and gastrocnemius muscles of the dominant leg during treadmill walking. Maximal voluntary isometric contractions while sitting were used for normalisation of sEMG to % of Maximum Voluntary EMG (%MVE). Knee joint angles during treadmill walking were measured by electrogoniometer. Furthermore, co-contraction index (CCI) was calculated, and presented for muscle groups of hamstrings-quadriceps (HQ) and gastrocnemius-quadriceps (GQ). CCI of medial and lateral sides of the knee, including ratio of the medial and lateral CCI for HQ and GQ were calculated.

**Results:**

No group differences were found in demographics, muscle activation level, nor CCI and CCI ratios. However, participants with GJH displayed significantly decreased knee joint angle, mean (153º vs. 156º; *p* =0.03) and minimum (105º vs. 111º; *p*=0.01), during treadmill walking compared with controls.

**Conclusion:**

Muscle activity during gait was not different between participants with GJH and non-GJH participants. However, participants with GJH displayed minor but statistically significant increased knee flexion during gait. Since the clinical consequences of increased knee joint flexion during gait are unknown, future studies should follow a larger cohort longitudinally during overground walking for development of clinical complications in this group.

## Background

Generalised Joint Hypermobility (GJH) is described as a condition where the joints are able to extend beyond the normal range of movement [1]. Prevalence varies across ethnic groups and is most frequent among females [2]. It is classified clinically with the Beighton score [3, 4], with a cut-off point of 6/9 for Caucasian children [5, 6]. Using these cut-off points, a recent study has estimated the prevalence of GJH to represent about 3–6 % in an Australian normal population [7].

GJH is a condition which for many is asymptomatic, while for some it may be symptomatic and possibly a feature of an underlying genetic disorder with systemic manifestations. When GJH becomes symptomatic, pain is a key factor in both children [8–10], and adults [11] and the condition may in some cases be diagnosed as the more severe Hypermobility Spectrum Disorder (HSD) [12], or an inherited connective tissue disorder, like hypermobile Ehlers-Danlos Syndrome (hEDS).

Musculoskeletal function has been investigated in children and adolescents with GJH, with mixed results. Altered muscle activation pattern was seen in 10–15 years old children with GJH during standardized tests, such as isometric knee joint flexion [13, 14], static balance tasks [15], and in one-legged hop for distance [16]. During these tests, muscle activity was lower in the hamstring muscles [13, 14, 16], and muscle co-contraction [15, 16] or co-activation [13, 14] was increased, which may be seen as compensatory strategies to improve joint stability of the hypermobile joints. Whether such changed muscle activity is present also during gait is still unknown.

Previous research has demonstrated differences in gait kinematics and kinetics between children with and without GJH, including decreased peak knee flexion and increased knee extension [17], larger range of motion in the ankle and hip [9], lower knee and hip joint moments [18], as well as altered strategies for trunk stability [19]. Altered gait biomechanics has been suggested to be a risk factor for osteoarthritis in the lower limb joints later in life [20, 21]. However, the underlying neurophysiological mechanisms for altered gait kinetics and kinematics in participants with GJH are unknown.

Thus, the aim of the present study was to investigate potential group differences in knee muscle activity simultaneously with knee joint kinematics during treadmill walking. Based on previous results on isometric contractions, our hypothesis was that girls with GJH would display different knee muscle activity, seen as decreased hamstrings activity and increased co-contraction, besides decreased peak knee flexion and increased knee extension, compared with girls without GJH (non-GJH) during walking.

## Methods

### Participants

The study was a case-control study. Participants were recruited from the Copenhagen Hypermobility Cohort (COHYPCO), a cohort of Caucasian schoolchildren where the prevalence of GJH was investigated [22–24]. The COHYPCO consists of 354 children with and without GJH from 18 public schools in 2 midsize Danish municipalities in a rural area of Greater Copenhagen. Adolescent girls (14–15 years of age) with GJH were recruited from COHYPCO, and a randomly selected age-matched control group (the first 10 girls on the class list who accepted participation and met the inclusion criteria for the non-GJH group) was recruited from non-GJH classmates in the cohort.

Inclusion criteria for the GJH-group based on data from COHYPCO were: girls with a Beighton score ≥ 6 and a positive score for hyperextension ≥ 10° in the dominant knee or both knees, while for the non-GJH group, it was girls with a Beighton score ≤ 5 and no knee hyperextension, measured during static standing (Table 1). Exclusion criteria for both groups were previous or current knee pain, serious/severe trauma of the lumbar spine or lower extremities, and hereditary diseases like Ehlers–Danlos Syndrome, Marfan Syndrome or Osteogenesis Imperfecta, in addition to inability to understand and speak Danish [15]. A total of 68 girls from the cohort met the eligibility criteria and were invited to participate (GJH = 28, non-GJH = 40). Twenty-six girls accepted the invitation and fulfilled the inclusion criteria (16 participants with GJH and 10 non-GJH participants). Participants of COHYPCO were clinically re-examined according to the Beighton criteria prior to participation in the present study to ensure that they fulfilled the inclusion criteria.

**Table 1:** Beighton Score [3, 4]

Clinical manoeuvre	Unable to perform	Able to perform
Thumbs to the volar aspect of the forearm with extended elbow and pronated hand		
Left	0	1
Right	0	1
5th finger dorsiflexion 90^o^ with forearm and wrist flat on the table		
Left	0	1
Right	0	1
Elbow extension >10^o^ with arms abducted at the shoulder and hand supinated		
Left	0	1
Right	0	1
Knee extension >10^o^ in standing position, foot on the floor		
Left	0	1
Right	0	1
Forward bending of the trunk with straight legs, hands flat on the floor	0	1

Maximum Beighton score is 9 points

The parents and girls were informed about the aims of the study. The parent of each participating girl gave informed written consent to participate, and each girl gave oral consent to participation prior to testing. All procedures were performed according to the Declaration of Helsinki. The Committee on Biomedical Research Ethics for the Capital Region of Denmark approved the study (H-4-2012-150).

### Protocol

#### Demographics

All participants were asked to answer questions on sports participation (yes/no) and the weekly number of hours of sports participation. Leg dominance was determined as the leg used for a minimum of two of the following tasks: (i) the leg used to step up on an electronic scale (tested), (ii) the preferred leg in a balance response (tested), and (iii) the leg used for kicking a ball (self-reported).

#### Maximal voluntary contractions

The experimental setup was the same as described in earlier publications [13–15] with satisfactory psychometric properties [25]. In short, the girls were seated in a chair with the thigh fixed into a horizontal position with Velcro straps. The lower leg was fixed with a strap (force transducer inserted) at level of the ankle. In this position, the girls performed maximal voluntary isometric knee flexion and extension over five seconds with a fixed knee angle of 90°. Three repetitions were performed in each direction, separated by a minimum of 1 min of rest between each contraction to avoid fatigue. If the last performed contraction was the highest force recorded, an additional repetition was performed to ensure that maximum force was measured. A maximum of two additional contractions were performed to avoid fatigue. The contraction with the highest exerted force was selected as maximal voluntary contraction (MVC). Familiarisation was performed before measurements. The order of flexion contractions and extension contractions were randomised between participants.

#### Treadmill walking

Participants performed level walking (unshod) at 4 km/h on a motorised treadmill for 5 min, with their gaze fixed onto a single spot at the eye height of each participant approximately 1.5 m in front of the participant. A walking speed of 4 km/h is expected to be close to preferred walking speed [26, 27]. The first two minutes of walking were considered familiarization and warm-up, and data were sampled during the last three minutes (180 s) with no speaking or irregularities allowed, such as turning the head. Data from the first and last 30 sec of the measured gait cycles were excluded from the analyses, resulting in 120 s analysis of gait cycles. All data presented is based on recordings of the entire gait cycle.

### Measurements

#### EMG

EMG was used for measuring muscle activity and has been tested to have satisfactory psychometric properties [28, 29]. Bipolar surface electromyography (sEMG) was recorded from the dominant leg (Ag/AgCl electrodes, 720-01-K, Medicotest, Denmark). According to current recommendations (www.seniam.org) two electrode pairs were placed at the knee extensor muscle m. quadriceps femoris (Q) corresponding to vastus lateralis (VL) and vastus medialis (VM), and four electrode pairs at the knee flexor muscles and the hamstrings muscles (H), corresponding to m. biceps femoris (BF), m. semitendinosus (ST) and m. gastrocnemius muscles (G) including the lateral (GL) and medial head (GM). The reference electrode was placed at the tibial bone [15].

The electrodes were attached to the skin with an inter-electrode centre distance of 2 cm, in line with the muscle fibre orientation [30]. The skin was prepared by shaving any body hair, abrading the skin with sandpaper, and then cleansed with alcohol before attachment of the electrodes to obtain an inter-electrode resistance < 10 KOhm.

The EMG signals were pre-amplified (×25), filtered with a band-pass of 10–400 Hz (Logger Technology, Aakarp, Sweden) and sampled at a sampling rate of 1 kHz (DT BNC Box, USB 9800 series, Data Translation, Marlborough, MA, USA and Scope, Version 2.3, Data Translation, Germany).

#### Electrogoniometer

An electrogoniometer (Penny and Giles, Angle Display Unit. Model SG-110, Biometrics Ltd., UK) was placed on the lateral side of the knee with the knee joint centre in the middle of the goniometer and trochanter major at the femur and the lateral malleolus of fibula as anchor points. The electrogoniometer was attached when the participants were standing (knee joint angle ~ 180°, knee extension ~ 0°) and fastened with fixomull medical tape. The knee joint angle was measured as the angle between femur and tibia in the sagittal plane, representing knee flexion/extension movements. Thus, the fully extended knee joint corresponded to a knee angle of approximately 180°, and a knee extension angle of approximately 0°.

The electrogoniometer, was calibrated at known fixed angles of 90° and 180° to determine V/° (the voltage (V) output from the electrogoniometer at each of the measured knee joint angles (°)). After attachment of the electrogoniometer, the precise placement of the goniometer was measured at knee joint angles of 90° and 180° using a manual goniometer for calibration to precisely determine the V/° of each participants leg. The electrogoniometer used in this study is considered very accurate with measurement errors between 1.0º and 2.0º [31]. The same period during the gait task as for the EMG was recorded (sampling rate 1 kHz) and used for analyses of the gait cycles.

#### Data management and processing

Data were analysed with EMGview (EMGview 1.3, Laursen, Denmark). RMS (Root mean square) values of each 100-msec segment were calculated and normalized to %MVE (% Maximal Voluntary Electromyography) for each participant. MVE was calculated as 1-sec mean values based on 10 successive 100-msec RMS values. EMG measured during gait were high-pass filtered at 5 Hz with a dual-pass (forward-backwards) zero lag 4th order Butterworth filter in order to remove potential movement artefacts.

Co-contraction index (CCI) was calculated as the average of consecutive instantaneous muscle activity ratios of a co-contracting muscle pair multiplied by the instantaneous summed muscle activity of the co-contracting muscle pair [32].

$$ CCI=\frac{\left[{\sum}_{i=1}^n\ \frac{\%{MVE}_{\mathit{\min},i}}{\%{MVE}_{\mathit{\max},i}}\ast \left(\%{MVE}_{\mathit{\min},i}+\%{MVE}_{\mathit{\max},i}\right)\right]\ }{n} $$

The less activated muscle is equal to ‘min’, and ‘max’ is equal to the more activated muscle. CCI’s were calculated for the muscle pairs HQ (hamstrings-quadriceps) and GQ (gastrocnemius-quadriceps), and for the medial and lateral sides of the knee, respectively, presented as the medial CCI and the lateral CCI of HQ (semitendinosus to vastus medialis and biceps femoris to vastus lateralis), and likewise for GQ (gastrocnemius medialis to vastus medialis and gastrocnemius lateralis to vastus lateralis). In addition, ratios between the medial and the lateral CCI for HQ and for GQ were calculated.

The calculated knee joint angles during treadmill walking for the two groups were; mean knee joint angle during gait (°) across the entire gait cycle, maximum knee joint angle for each gait cycle (°), minimum knee joint angle for each gait cycle (°) and mean knee joint range of movement (ROM, Δ°).

### Statistical analyses

A Kolmogorov-Smirnov test was used to test for normal distribution of the different variables. An unpaired student’s t-test, two-tailed assuming equal variances, was used to test for group differences (GJH and non-GJH) on demographic variables. Further, a general linear regression model (GLM, mixed model) was performed, using variables for EMG, CCI and knee joint angles as dependent factors (one at a time), while status (GJH/non-GJH) and weekly sport participation (yes/no) were fixed factors in the model. EMG and knee joint angles are reported as group average values followed by SD.

All statistical analyses were performed in the software SPSS Statistics (IBM SPSS Statistics, version 25, 2017). Statistical significance was defined as *p* ≤ 0.05.

## Results

In total, 26 children were included in the analyses, corresponding to 16 participants with GJH and 10 non-GJH participants. Due to technical errors, kinematics data are missing for one GJH participant, leaving 15 girls in the GJH group for analysis. The groups were comparable on demographics, except for the Beighton score where participants with GJH had a higher score than non-GJH participants (GJH: 6.8 (0.7), median 7, range 6–8; non-GJH: 1.3 (1.6) median 1, range 0–5 (*p* < 0.01)) (Table 2).

**Table 2:** Demographics (mean (SD), n (%)) for girls with Generalized Joint Hypermobility (GJH) and girls without (non-GJH)

	GJH (*n* = 16)	non-GJH (*n* = 10)	*p*-value
Beighton score (0–9), mean (SD)	6.8 (0.7)	1.3 (1.6)	< 0.01*
Age (years), mean (SD)	14.0 (0.0)	14.3 (0.5)	0.019*
BMI (kg/m^2^), mean (SD)	21.0 (2.8)	21.0 (1.9)	0.971
Height (cm), mean (SD)	165.8 (4.9)	165.8 (4.4)	0.968
Weight (kg), mean (SD)	57.7 (9.1)	57.7 (4.7)	0.986
Sports weekly (yes), n (%)	12 (75)	9 (90)	0.617
Hours/week with sports, mean (SD)	3.7 (3.8)	4.1 (3.0)	0.754
Knee pain on the test day, n (%)	1 (6)	1 (10)	1.000

*indicates a statistically significant difference (*p-value* < 0.05) between participants with GJH and non-GJH

The participants in the GJH group displayed significantly lower mean knee joint angle, corresponding to (SD) (152.7º (4.5) vs. 156.3º (2.1); *p* = 0.03) and minimum knee joint angle (104.9º (5.1) vs. 110.5º (2.9); *p* = 0.01) during treadmill walking, meaning significantly increased knee joint flexion in GJH during gait (Table 3). There were neither group differences in mean muscle activity (Table 4), nor in CCI and CCI ratios between the medial and lateral muscle groups (Table 5).

**Table 3:** Knee joint angle (mean, SD) during treadmill walking for girls with Generalized Joint Hypermobility (GJH) and girls without GJH (non-GJH). 180° corresponds to the knee joint being extended

	GJH (*n* = 15)	non-GJH (*n* = 10)	*p*-value
Mean (°)	152.7 (4.5)	156.3 (2.1)	0.03*
Max (°),	177.8 (6.0)	180.7 (3.7)	0.19
Min (°),	104.9 (5.1)	110.5 (2.9)	0.01*
ROM (Δ°),	72.8 (5.3)	70.1 (5.6)	0.24

*indicates a statistically significant difference (*p-value* < 0.05) between participants with GJH and non-GJH

**Table 4:** Electromyography (EMG) in %MVE (mean, SD) measured during treadmill walking for girls with Generalized Joint Hypermobility (GJH) and girls without GJH (non-GJH)

	GJH (*n* = 16)	non-GJH (*n* = 10)	*p*-value
Quadriceps (Q)	4.9 (2.0)	5.0 (1.5)	0.99
Vastus lateralis (VL)	5.5 (2.8)	5.3 (1.4)	0.78
Vastus medialis (VM)	4.4 (1.7)	4.7 (2.0)	0.68
Hamstrings (H)	6.3 (2.4)	6.7 (2.6)	0.60
Biceps femoris (BF)	5.5 (2.3)	6.3 (2.9)	0.39
Semitendinosus (ST)	7.1 (3.1)	7.1 (2.9)	0.91
Gastrocnemius (G)	11.7 (3.4)	12.3 (4.9)	0.77
Gastrocnemius lateralis (GL)	9.7 (3.6)	11.2 (5.2)	0.34
Gastrocnemius medialis (GM)	13.6 (4.6)	13.5 (5.8)	0.80

*MVE* maximal voluntary electromyography, *Q *quadriceps, *VL *vastus lateralis, *VM *vastus medialis, *H *hamstrings, *BF *biceps femoris, *ST *semitendinosus, *G* gastrocnemius, *GL *gastrocnemius lateralis, *GM *gastrocnemius medialis

**Table 5:** Co-contraction index (CCI) (mean, SD) during treadmill walking for girls with Generalized Joint Hypermobility (GJH) and girls without GJH (non-GJH)

	GJH (*n* = 16)	non-GJH (*n* = 10)	*p*-value
Mean HQ	4.4 (1.3)	5.4 (1.9)	0.14
Medial HQ (ST,VM),	4.1 (1.4)	4.9 (2.3)	0.27
Lateral HQ (BF,VL),	3.8 (1.1)	4.7 (1.6)	0.12
Ratio medial/lateral HQ	1.1 (0.3)	1.1 (0.4)	0.68
Mean GQ	4.2 (1.9)	4.0 (1.4)	0.79
Medial GQ (GM,VM),	3.5 (1.7)	3.6 (1.8)	0.96
Lateral GQ (GL,VL),	4.3 (2.1)	4.0 (1.1)	0.77
Ratio medial/lateral GQ	0.9 (0.3)	0.89 (0.3)	0.87

*HQ *hamstrings-quadriceps, *GQ *gastrocnemius-quadriceps, *Q *quadriceps, *VL *vastus lateralis, *VM *vastus medialis, *H *hamstrings, *BF *biceps femoris, *ST *semitendinosus, *G *gastrocnemius, *GL *gastrocnemius lateralis, *GM *gastrocnemius medialis

## Discussion

The GJH group showed a minor but statistically significant decreased knee joint angle indicating increased knee joint flexion during treadmill walking compared to non-GJH participants. However, the participants with GJH had neither altered relative muscle activation nor CCI and CCI ratios compared with non-GJH participants.

The current data on relative muscle activation seems, at first, to be in contrast with some of the previous studies on muscle activity in children with GJH (10 years and14 years, Beighton ≥5 or ≥6), where reduced hamstrings activity and increased co-activation/CCI during tasks, such as maximal and submaximal isometric knee extension/flexion while sitting [13, 14], were found. It has been suggested that increased co-activation/CCI is a compensatory strategy to stabilize the knee joint in flexed positions [13]. Furthermore, previous studies have shown that the reduced hamstrings activity found in participants with GJH is less distinct in more extended knee positions when measured in 90, 110 and 130 degrees, as hamstrings activity increases with increased knee extension and oppositely decrease with more pronounced knee flexion when compared to non-GJH participants [14]. The current study does not display lower, but the same level of muscle activity of the hamstrings muscles in participants with GJH and in the non-GJH participants during treadmill walking where the knee joint angles correspond largely to ‘extended knee positions’ during gait. Thus, the current results support previous results [14] indicating no differences in muscle activation between participants with GJH and non-GJH participants in extended knee joint positions.

One study, though, found a significantly higher activity in quadriceps and hamstrings muscles during overground gait in young females with GJH compared with a matched group of non-GJH [33]. However, overground walking may differ from treadmill walking, as found previously on muscle activity in healthy persons, where treadmill walking showed higher rectus femoris activity in e.g. the transition from stance to swing and higher hamstrings and vastus medialis activity at terminal swing. Oppositely the hamstrings and vastus medialis activity was lower through the early and mid-swing phase, whereas very few differences were found in leg kinematics and temporal gait parameters [34]. The lack of between-group differences in the current muscle activity and CCI may, therefore, be explained by the different characteristics of the gait task (the current standardized walking speed on the treadmill vs. self-selected speed in over ground gait [34]). Other reasons may be differences in data presentation (the current mean values of the whole gait cycle vs. specific phases of the gait cycle [35]), differences in participant characteristics (the current non-GJH and GJH non-symptomatic vs. non-GJH and a mix of symptomatic and non-symptomatic GJH, as well as adolescents vs. adults). However, muscle activation patterns in the studies look very similar, and the actual differences in muscle activity were very small. Furthermore, the authors have questioned the clinical relevance of such small group difference in muscle activity between the group with and without GJH [33].

With respect to knee joint angles during gait, previous studies show diverging results. The current data on girls with non-symptomatic GJH show increased knee joint flexion during gait (in the swing phase), in line with a study of adults with non-symptomatic GJH (during stance phase) [36]. In contrast, increased knee joint extension during gait was seen in children with symptomatic GJH, e.g. Hypermobility Syndrome [9, 17], while no differences between non-symptomatic GJH and non-GJH were seen in 10-year old children [18] and adults with EDS hypermobile type (symptomatic) [37]. The reason for these differences, however, is unclear as comparisons are difficult to perform since either results on means of the entire gait cycle or means of specific periods (like heel strike or peak knee flexion) are presented. Furthermore, the present group difference showed considerable variation and was only about 3º (GJH: 152.7 (4.5) º and non-GJH: 156.3 (2.1) º) for mean knee joint angle and 6º (GJH: 104.9 (5.1) º and non-GJH: 110.5 (2.9) º) for minimum knee joint angle. Therefore, the clinical relevance of the small detected differences is still unclear, and longitudinal studies are therefore needed to determine potential long-time consequences of these differences. Further, it is essential to note that the current group with GJH represents a non-symptomatic condition and not an actual diagnosis, and the long-term consequences of this condition are unknown concerning symptom development.

One limitation of this study is the small sample size. We do not think that the relatively small sample size has biased our results, since group differences in muscle activity and CCI/Co-activation in this group has been found previously with the same sample size. Despite many statistical comparisons we do not consider risk of type 1 error to be large, since all knee joint angle analyses pointed in the same direction, showing GJH to have larger knee joint flexion/smaller angles and smaller ROM than non-GJH, of which two of these were significant and supported each other. Further, lack of blinding of the status of GJH/non-GJH during the measurement is a limitation, but since objective measurements were used, this is not likely to have biased the results. Also, a selected number of knee muscles was measured, but it remains unknown whether measurements of additional muscles may have shown group differences. Lastly, measuring only sagittal plane knee motion is a limitation.

The strengths of this study are the comparable groups on demographic variables, except for the pre-defined status of GJH. Further, the use of standardized methods for classifying participants into GJH/non-GJH (Beighton tests), besides the standardized protocols for sEMG and electrogoniometer measurements are strengths of the study.

## Conclusions

Girls at 14–15 years with GJH have minor but statistically significant lower mean and minimum knee joint angles (increased knee joint flexion) during treadmill walking than a matched group without GJH. However, there were no group differences in surface EMG activity in the quadriceps, hamstrings and gastrocnemius muscles. Neither was there a group difference in co-contraction index (CCI) for any of the muscle groups or for ratios between CCI of the medial and lateral muscle groups.

Since the clinical consequences of increased knee joint flexion during gait are unknown, future studies should follow a larger cohort longitudinally during overground walking for development of clinical complications in this group.

## Data Availability

The datasets from the current study are available from the authors on reasonable request.

## Abbreviations

GJH: Generalised Joint Hypermobility
EDS: Ehlers–Danlos syndrome
Q: m. quadriceps femoris corresponding to vastus lateralis (VL) and vastus medialis (VM)
H: hamstrings corresponding to m. biceps femoris (BF), m. semitendinosus (ST)
G: m. gastrocnemius, lateral head (GL) and medial head (GM)
CCI: Co-contraction index
MVE: Maximal voluntary electromyography
sEMG: Surface electromyography
